# DRAMs and autophagy: a family affair

**DOI:** 10.1080/27694127.2022.2062965

**Published:** 2022-04-28

**Authors:** Valentin J. A. Barthet, Kevin M. Ryan

**Affiliations:** a Cancer Research UK Beatson Institute, Garscube Estate, Switchback Road, Glasgow, G61 1BD, UK; b Institute of Cancer Sciences, University of Glasgow, Garscube Estate, Switchback Road, Glasgow, G61 1QH, UK; c Cancer Biology and Genetics Program, Memorial Sloan Kettering Cancer Center, New York, NY, USA

**Keywords:** Autophagy, cell survival, DRAM family, DRAM4, DRAM5, nutrient deprivation

## Abstract

Macroautophagy/autophagy is a conserved catabolic process that delivers intracellular materials to lysosomes for degradation. This process promotes cell survival by maintaining cellular homeostasis and supplying substrates to produce energy during nutrient-deprived conditions. DRAM1 (DNA damage regulated autophagy modulator 1) is involved in DNA-damage-induced autophagy and belongs to an evolutionarily conserved protein family, which contains five members in human. Our recent study provides the initial characterization of the last two members of the DRAM family in autophagy regulation. Our findings revealed that TMEM150C/DRAM4 (transmembrane protein 150C) and TMEM150A/DRAM5 are nutrient-responsive members of the DRAM family that manifest interconnected roles in modulating the autophagic flux and cell survival under nutrient-deprived conditions.

**Abbreviations:**

DRAM1: DNA damage regulated autophagy modulator 1 EBSS: Earle’s balanced salt solution MAP1LC3/LC3: Microtubule associated protein 1 light chain 3

Macroautophagy (hereafter referred to as autophagy) is an intracellular recycling process that maintains homeostasis. While different members of the DRAM family have been described to modulate autophagy and/or cell survival, two remaining members of this family (TMEM150C/DRAM4 and TMEM150A/DRAM5) remained uncharacterized as to their role in autophagy and cell survival.

To determine the factors inducing TMEM150C/DRAM4 or TMEM150A/DRAM5 expression, we evaluated whether TMEM150C/DRAM4 and TMEM150A/DRAM5 are TP53/p53 targets like DRAM1. However, like DRAM2 and TMEM150B/DRAM3, we found that TMEM150C/DRAM4 and TMEM150A/DRAM5 are not induced by TP53, nor by DNA-damaging agents (cisplatin or etoposide). In addition, TMEM150C/DRAM4 and TMEM150A/DRAM5 are not induced in response to inflammatory stimuli (TNF or IFNG/IFNγ) unlike DRAM1. In contrast, we observed that TMEM150C/DRAM4 and TMEM150A/DRAM5 are induced by nutrient deprivation. TMEM150C/DRAM4 expression is induced upon serum deprivation or amino acids and serum-deprived conditions, whereas TMEM150A/DRAM5 expression is induced upon glucose or amino acids and serum (EBSS) deprivation. The induction of TMEM150C/DRAM4 and TMEM150A/DRAM5 expression by nutrient deprivation differs in a cell line-specific manner.

hDRAM family members localize to lysosomes (DRAM1, DRAM2, and TMEM150B/DRAM3), endosomes (DRAM2 and TMEM150B/DRAM3), and actin-rich focal adhesions at the plasma membrane (TMEM150B/DRAM3). We investigated whether TMEM150C/DRAM4 or TMEM150A/DRAM5 localizes to similar cellular compartments as other members of the DRAM family. We found that TMEM150C/DRAM4 is expressed in endosomes (early endosomes), whereas TMEM150A/DRAM5 localizes to adherens junctions at the plasma membrane. These results indicate that if DRAM family members have similar functions, then they potentially execute these functions at different locations within the cell.

To assess the role of TMEM150C/DRAM4 and/or TMEM150A/DRAM5 in modulating autophagy, we overexpressed either TMEM150C/DRAM4 or TMEM150A/DRAM5 in a human osteosarcoma cell line (Saos2), which expresses an average level of *TMEM150C/DRAM4* and *TMEM150A/DRAM5* mRNA. We observed that overexpression of TMEM150C/DRAM4 or TMEM150A/DRAM5 leads to a significant increase of LC3-II levels in EBSS. To distinguish between the possibilities that LC3-II accumulation upon TMEM150C/DRAM4 or TMEM150A/DRAM5 overexpression results from autophagy flux induction or autophagy impairment at a later stage following LC3-II formation, we treated cells with the lysosomotropic agent chloroquine, which blocks the turnover stage of autophagy. Following chloroquine treatment, we determined that TMEM150A/DRAM5 or TMEM150C/DRAM4 overexpression leads to an increase or a decrease in autophagy flux, respectively. These results demonstrate that TMEM150A/DRAM5 promotes autophagy, whereas TMEM150C/DRAM4 impedes autophagy at a stage post LC3-II formation (Figure 1).

**Figure 1.**
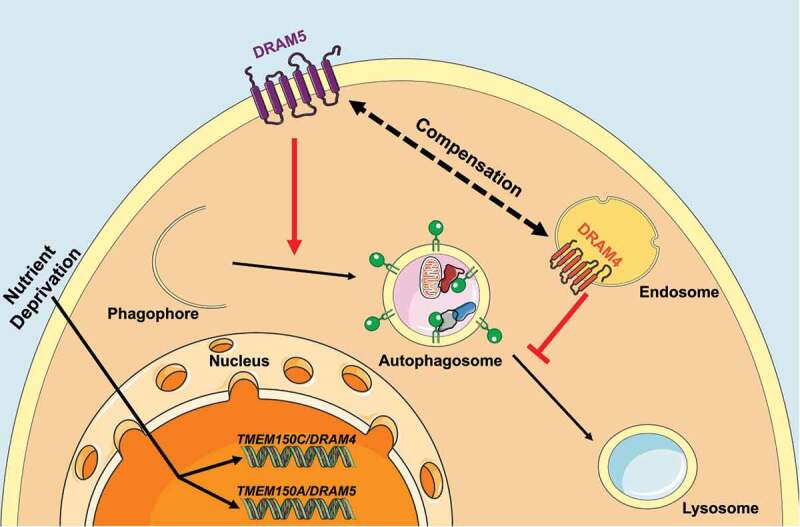
Graphical representation of the role of TMEM150C/DRAM4 and TMEM150A/DRAM5 in autophagy. TMEM150C/DRAM4, localizes at endosomes and impedes autophagy at a stage after autophagosome formation. In contrast, TMEM150A/DRAM5, localizes at the plasma membrane and promotes autophagic flux. Both TMEM150C/DRAM4 and TMEM150A/DRAM5 are induced upon nutrient deprivation.

To complement our overexpression findings, we next assessed whether reducing the expression of TMEM150C/DRAM4 or TMEM150A/DRAM5 modulates autophagy. We first determined that *TMEM150C/DRAM4* and *TMEM150A/DRAM5* are significantly expressed in different breast cancer cell lines, with MDA-MB-157 cells expressing a high level of both *TMEM150C/DRAM4* and *TMEM150A/DRAM5*. Following CRISPR-Cas9-mediated knockout of *TMEM150C/DRAM4* or *TMEM150A/DRAM5* in MDA-MB-157 cells, we observed that *TMEM150A/DRAM5* deletion does not affect LC3-II levels under basal conditions or upon EBSS starvation. In contrast, *TMEM150C/DRAM4* deletion increases LC3-II levels, therefore promoting autophagy flux, and, consequently, autophagy.

As TMEM150C/DRAM4 overexpression blocks autophagy at a stage post formation of autophagosomes, it was surprising for us that *TMEM150C/DRAM4* deletion results in induction of autophagic flux. Because TMEM150A/DRAM5 promotes autophagy flux and TMEM150C/DRAM4 and TMEM150A/DRAM5 share a high peptide sequence similarity, we hypothesized that TMEM150A/DRAM5 compensates for the loss of *TMEM150C/DRAM4*. To test this, we assessed *TMEM150A/DRAM5* gene expression upon loss of *TMEM150C/DRAM4*. We found that *TMEM150A/DRAM5* expression is upregulated following *TMEM150C/DRAM4* loss. Of note, we also demonstrated that this compensatory mechanism is reciprocal with *TMEM150A/DRAM4* expression increasing upon *TMEM150A/DRAM5* loss.

This compensatory mechanism between TMEM150C/DRAM4 and TMEM150A/DRAM5 led us to investigate whether the increase in autophagy flux we observed upon *TMEM150C/DRAM4* loss was due to TMEM150A/DRAM5 upregulation, which we described earlier as an autophagy flux inducer when TMEM150A/DRAM5 expression is increased ectopically. To test this, we generated a *TMEM150C/DRAM4 TMEM150A/DRAM5* double-knockout in MDA-MB-157 cells. We found that double-knockout cells do not increase LC3-II levels in basal conditions or upon EBSS starvation. These findings confirm the compensatory role between TMEM150C/DRAM4 and TMEM150A/DRAM5, and demonstrate that *TMEM150C/DRAM4* loss induces autophagy through TMEM150A/DRAM5 upregulation.

Autophagy promotes survival in response to nutrient deprivation. Because TMEM150C/DRAM4 and TMEM150A/DRAM5 are induced in nutrient-deprived conditions and modulate autophagy, we hypothesized that TMEM150C/DRAM4 and/or TMEM150A/DRAM5 regulate cell survival when nutrients are restricted. To evaluate this, we challenged MDA-MB-157 cells knocked out for *TMEM150C/DRAM4, TMEM150A/DRAM5*, or both *TMEM150C/DRAM4* and *TMEM150A/DRAM5* under different nutrient-deprived conditions and assessed their clonogenic potential. We observed that *TMEM150C/DRAM4* loss promotes survival in the absence of serum, glucose, and amino acids and serum (EBSS) in a TMEM150A/DRAM5-dependent manner.

In summary, we report the initial characterization of TMEM150C/DRAM4 and TMEM150A/DRAM5 in comparison to the other members of the DRAM family [1]. We demonstrate that TMEM150C/DRAM4 and TMEM150A/DRAM5 are not induced by TP53, but rather by nutrient-deprived conditions like DRAM2 and TMEM150B/DRAM3. As with other members of the DRAM family, TMEM150C/DRAM4 and TMEM150A/DRAM5 regulate autophagy. Although TMEM150C/DRAM4 impedes autophagy after autophagosome formation, and TMEM150A/DRAM5 promotes autophagosome biogenesis; protein-protein interaction studies are needed to elucidate their interacting partners in modulating autophagy. The compensation mechanism between TMEM150C/DRAM4 and TMEM150A/DRAM5 on autophagy function is not surprising considering their sequence similarity; however, it raises the question of how this compensatory regulation operates, because both proteins are expressed in different cell compartments. According to their expression in breast cancer cell lines and their function in cell survival, generating mice knocked out for *Tmem150c/Dram4* and/or *Tmem150a/Dram5* would be beneficial to evaluate the impact of both genes in breast cancer *in vivo*, and potentially in other diseases.
